# Molecular Genetics of Follicular-Derived Thyroid Cancer

**DOI:** 10.3390/cancers13051139

**Published:** 2021-03-07

**Authors:** Elisabetta Macerola, Anello Marcello Poma, Paola Vignali, Alessio Basolo, Clara Ugolini, Liborio Torregrossa, Ferruccio Santini, Fulvio Basolo

**Affiliations:** 1 Department of Surgical, Medical, Molecular Pathology and Critical Area, University of Pisa, via Savi 10, 56126 Pisa, Italy; elisabetta.macerola@for.unipi.it (E.M.); marcellopoma@gmail.com (A.M.P.); paola.vignali@phd.unipi.it (P.V.); clara.ugolini@unipi.it (C.U.); l.torregrossa@ao-pisa.toscana.it (L.T.); 2 Department of Clinical and Experimental Medicine, University of Pisa, via Savi 10, 56126 Pisa, Italy; alessio.basolo@med.unipi.it (A.B.); ferruccio.santini@med.unipi.it (F.S.)

**Keywords:** molecular genetics, molecular pathology, thyroid cancer, papillary thyroid cancer

## Abstract

**Simple Summary:**

Thyroid tumors that derive from follicular cells are not a homogeneous entity, showing variable morphological appearance and different degrees of differentiation. Molecular markers are useful for both diagnostic purposes and prognostic stratification of patients. In presurgical setting, molecular testing of indeterminate thyroid nodules on aspirates provides useful diagnostic information; the molecular analysis on tumor tissues can also reveal the presence of genetic alterations related to patients’ prognosis. In recent years, the molecular characterization of these tumors has acquired even more importance thanks to the introduction of targeted drugs. This review summarizes the current literature on the molecular landscape of follicular-derived thyroid tumors.

**Abstract:**

Thyroid cancer is the most common type of endocrine-related malignancy, whose incidence rates have increased dramatically in the last few decades. Neoplasms of follicular origin generally have excellent prognosis, with the exception of less differentiated tumors. Follicular-derived thyroid cancer can manifest as a variety of morphologically distinct entities, characterized by various degrees of differentiation and invasiveness. Histological evaluation is thus crucial for the definition of patients’ prognosis. However, within each histological subtype, tumor behavior can be highly variable, and, in this respect, molecular characterization can provide insightful information to refine the risk stratification of tumors. In addition to the importance of its prognostic role, molecular testing can be used to support the differential diagnosis of thyroid nodules in the absence of marked cyto-morphological aberrations. Finally, with the advent of targeted drugs, the presence of molecular alterations will guide the therapeutic strategies for patients with advanced tumors who do not respond to standard treatment. This review aims to describe the genetic landscape of follicular-derived thyroid tumors also highlighting differences across histological subtypes.

## 1. Introduction

Thyroid tumors originated from follicular cells, namely non-medullary thyroid cancers, encompass 95% of all thyroid malignancies [1]. A primary gross distinction can be made between well-differentiated thyroid carcinomas (WDTC), which include papillary thyroid carcinomas (PTC), follicular thyroid carcinomas (FTC), and Hürthle cell thyroid carcinomas (HCC), and less differentiated forms, i.e., poorly differentiated thyroid carcinomas (PDTC) and anaplastic, or undifferentiated, thyroid carcinomas (ATC). PTC is the most common endocrine malignancy, accounting for approximately 85% of all follicular-derived thyroid cancers, while FTC occurs in less than 10% of all thyroid tumors [2]. In general, WDTC patients have excellent 10-year survival rates, but prognosis highly depends on molecular and clinico-pathological characteristics. For instance, the noninvasive follicular neoplasm with papillary-like nuclear features, NIFTP, is a borderline lesion introduced in 2016 from the reclassification of a specific, indolent subtype of PTC [3]. On the other hand, there are PTC variants, such as the hobnail variant, which often present with gross local invasion as well as lymph node and distant metastases, and has a high recurrence rate, therefore representing a non-negligible threat to patients’ survival [4].

The molecular hallmarks among and within histological subtypes can be highly variable and may impact on patients’ prognosis. In particular, the presence of secondary mutations defines a subgroup of aggressive tumors, which are often resistant to standard treatment [5]. In this context, targeted therapies have been emerging in the clinical management of thyroid cancer, in the light of which molecular tumor characterization will acquire even more significance. This review describes the molecular landscape of follicular-derived thyroid cancers, highlighting the differences among histological subtypes, with a particular focus on advanced tumors.

The most recent literature studies describing the presence of mutations and fusions in different types of thyroid tumors have been included in this review; in cases of availability of hundreds of studies (i.e., *BRAF* mutations in PTCs), massive-parallel sequencing-based studies and large sample size ones have been preferentially considered. With regard to advanced WDTCs, molecular studies on PTC with “aggressive variants” lacking clinical information were not considered.

The specific aim of this review is to provide a general overview of the most frequent gene mutations and gene fusions detectable in thyroid cancer, since these molecular alterations can be easily tested in all molecular pathology laboratories, and can also be therapeutically relevant. A short paragraph has been dedicated to other well-known and emerging markers helpful in the diagnostic and prognostic definition of these tumors.

## 2. Molecular Landscape of Well-Differentiated Thyroid Carcinoma (WDTC)

According to the definition provided by the World Health Organization (WHO) classification of tumors of endocrine organs [2], PTCs are malignant carcinomas with follicular differentiation showing a set of peculiar nuclear features and/or a papillary growth pattern. The nuclear changes of PTC can be summarized on the basis of three main categories: (1) size and shape: the nuclei may appear enlarged, elongated and overlapping; (2) nuclear membrane irregularities: the contours of the nuclei appear irregular; the nuclear grooves and pseudoinclusions are typically present; (3) chromatin changes: mainly presence of chromatin clearing and margination.

The typical appearance of PTC, the classical or conventional type (CPTC), includes the presence of both distinctive nuclear features and papillary pattern, with the tumor that is rarely totally encapsulated [2]. However, PTC appears in a wide range of microscopically distinct entities, the so-called PTC variants. Each variant does not only show peculiar histo-morphological and molecular characteristics, but can also assume a clinically different behavior.

After the classical type, the follicular (FVPTC) is the most frequent variant of PTC. It shows the papillary-like nuclear changes but has a predominant follicular growth pattern. FVPTC has probably been the most discussed histotype of all thyroid tumors over the last 20 years. This is presumably due to its highly variable biological, pathological and clinical manifestations. Three main groups of FVPTC can be distinguished on the basis of their features of invasiveness: noninvasive encapsulated, invasive encapsulated, and infiltrative [6]. The noninvasive encapsulated category has been frequently reported to be clinically indolent, to such an extent that a downgrading of this type of FVPTC—from “carcinoma” to “neoplasm”—has been proposed and obtained (see the NIFTP paragraph). A clear clinical/prognostic demarcation between encapsulated invasive and infiltrative FVPTC is hard to obtain because authors rarely make such a distinction. However, it seems that infiltrative FVPTC should be molecularly closer to CPTC [7].

The most frequent molecular alterations detected in PTCs occur in the MAP kinase (MAPK) signaling pathway, and mutations in the *BRAF* gene are found in 45–50% of all PTCs [8]. It is well-known that the prevalence of mutations varies according to the PTC variant, so that *BRAF*^V600E^ is detected in up to 70% of CPTCs, while its frequency is much lower in FVPTC, where it is found almost exclusively in the infiltrative forms [7]. The most frequent driver mutations detected in FVPTC, in up to 48% of cases, occur in the *RAS* family genes (*NRAS*, *HRAS* and *KRAS*) [7].

The Cancer Genome Atlas (TCGA) research network delivered a multiplatform extensive integrated molecular characterization of 496 PTCs, mainly classical and follicular variants [9]. This study highlights that, compared to other cancers, PTCs have a relatively stable genome with genetic mutations occurring or, better, recurring in a limited number of genes and a low mutation burden (about 0.41 non-silent mutations per megabase), so that more than one mutation rarely coexists in the same tumor. As expected, the most frequent driver mutation detected in PTC was BRAFV600E (57%), followed by point mutations in NRAS (8%) and RET fusions (7%). Mutations in *HRAS*, *EIF1AX*, *KRAS*, *PPM1D*, *CHEK2*, *BRAF* fusion, *PPARG* fusion and *THADA* fusion were cumulatively present in less than 5% of cases. *NTRK* fusions were also rare, being found in 2% of cases, although some authors had previously reported them in up to 3–5% of sporadic adult PTC [7,10]. It is worth noting that in thyroid cancer almost only *NTRK1* and *NTRK3* fusions have been described so far.

In addition to the fact that *BRAF*^V600E^ was prevalent in CPTC, while *RAS* mutations were enriched in FVPTC, the TCGA study has demonstrated that, by matching molecular results including genomic, epigenomic and proteomic data, two major categories of PTCs can be distinguished: the *BRAF*^V600E^-like and the *RAS*-like PTCs. *BRAF*^V600E^-like alterations include mutations other than the *BRAF*^V600E^ ones, as for example *RET/PTC* and *BRAF* fusions. These alterations are associated with a papillary architecture and a lower expression of thyroid-differentiation genes. On the contrary, *RAS*-like alterations (including *RAS* mutations, the *BRAF*^K601E^ mutation, *PPARG* fusions, *THADA* fusions and *EIF1AX* mutations) are associated with a follicular architecture and a close-to-normal expression of the genes related to thyroid differentiation [9]. The identification of this fundamental, deep distinction between CPTCs and FVPTCs has led many authors to wonder whether *BRAF*-like and *RAS*-like definitions could be extended to the other thyroid tumors.

For example, the tall cell variant PTC (TCVPTC, about 6% of all PTCs), an aggressive variant characterized by a high prevalence of *BRAF*^V600E^ mutation [11], could be easily placed among the *BRAF*^V600E^-like tumors. The same could be said for the hobnail variant PTC. On the other hand, for more rare PTC variants, dedicated genomic and transcriptomic studies are still necessary. For instance, the solid variant (1–3% of PTCs [2]) has been described to harbor not only *BRAF* mutations, but also *RET* and *NTRK* fusions; however, this variant seems to be molecularly different from CPTC [12].

FTCs, follicular-patterned tumors that lack PTC nuclear alterations, are usually encapsulated and show tumor capsule and/or vascular invasion. They represent 6–10% of follicular-derived thyroid tumors [2]. Unlike PTCs, which cause lymph node involvement in many patients, FTCs often show hematogenous spread to distant organs (mainly to the bones and lungs). The TCGA study results on PTC have been confirmed by several subsequent studies, where FTCs were also included [7,13,14]. Apart from the already known high prevalence of RAS (in up to 50% of cases) [15] and of *RAS*-like mutations in FTC, a genetic similarity has been demonstrated between FTCs and encapsulated FVPTCs [7].

Moreover, based on transcriptional data, the existence of a third-class of tumors has been hypothesized: the so-called Non-*BRAF*-Non-*RAS* (NBNR), which are neither *BRAF*-like, nor *RAS*-like; this class of tumors might also include *PPARG* fusions, and *EIF1AX* mutations, but these evidences need to be confirmed [7].

Finally, the HCCs are a group of encapsulated tumors predominantly composed of oncocytic cells and characterized by capsular/vascular invasion. The classification of these neoplasms as a subtype of follicular tumors has been a hotly-debated issue; according to the current indications of WHO, HCCs are independent histo-pathological entities belonging to the DTC [2]. HCCs account for 3–5% of all non-medullary thyroid tumors [16,17]. Similarly to FTC, HCCs show a higher incidence of distant metastases compared to PTCs. The molecular frame of HCCs is completely different from that of the rest of the WDTCs, being characterized by three main types of alterations: (1) mitochondrial DNA mutations, occurring as early events in genes encoding complex I subunits; (2) point mutations recurring in genes that are not typically mutated in thyroid cancer, with the exception of few *RAS* and *EIF1AX* mutations; (3) karyotype alterations, with tumors having a near-haploid state, a polysomic state and/or duplication of chromosomes 7, 5, and 12 [16,17].

Secondary mutations are not common in WDTCs. *TERT* promoter mutations show a frequency of about 10% in PTCs and 15% in FTCs [7,9,18]. However, a difference across PTC variants has been observed, with tall cell PTCs reaching a frequency of 25%. Similarly, *TERT* promoter mutations have been reported in 15–20% of HCCs, with a higher occurrence in widely invasive (32%), versus minimally invasive (5%) tumors [17,18]. Since *TERT* promoter mutations show a higher frequency in morphologically aggressive WDTCs such as tall cell PTCs and widely invasive HCCs, they can contribute in part to the evidence demonstrating their association with poor patient prognosis. Indeed, significant associations between *TERT* promoter mutations and distant metastases, disease persistence and recurrence, advanced stage and also patients’ survival have been demonstrated in WDTC patients [19,20,21]. The influence of *TERT* promoter mutations, mainly C250T and C228T substitutions, on prognosis can be attributed to the creation of de novo binding sites for transcription factors able to enhance the gene transcriptional activity and promote telomerase activation [21].

### Noninvasive Follicular Neoplasms with Papillary-Like Nuclear Features (NIFTP)

NIFTPs have been recently introduced in the scenery of thyroid histopathology. The advent of this follicular architecture lesion has followed decades of controversies on the evidence that noninvasive and well-encapsulated follicular variant PTCs show an extremely indolent clinical course. NIFTP is a well-encapsulated neoplasm that shows nuclear alterations typical of papillary carcinoma and a follicular architecture [3]. This tumor can be considered neither benign nor malignant, but rather a premalignant lesion. Due to the fact that NIFTP diagnosis requires histological examination, essential to assess the absence of invasive foci, the advent of NIFTP has mainly influenced the post-surgical setting, since completion thyroidectomy and radioiodine therapy are no longer required.

In an attempt to solve a long-standing debate, the introduction of NIFTP has led to other controversies, mainly related to its histological nature. As it can be diagnosed only after surgery, the cytological issue of indeterminate nodules (Bethesda III and IV) related to follicular-architecture thyroid lesions has been further complicated. The molecular frame of NIFTPs cannot help to solve this issue, since NIFTPs are *RAS*-like tumors, harboring *RAS* mutations in 40–70% of cases [22,23] in the same way as their invasive counterparts. NIFTPs should lack *BRAF*-like mutations, and in fact the presence of *BRAF*^V600E^ mutations has been proposed as an exclusion criterium for NIFTP diagnosis [24].

Secondary mutations in NIFTP have been rarely described, confirming that their morphologically and clinically indolent nature is accompanied by a relatively low-risk molecular profile.

## 3. Poorly Differentiated and Anaplastic Thyroid Carcinoma

Compared to WDTCs, PDTCs and ATCs are rare, accounting for 5–10% of all thyroid tumors, but their prognosis is much less favorable [2].

The diagnostic criteria for PDTC have been quite controversial. Two main systems are currently used: the Turin proposal and the Memorial Sloan Kettering Cancer Center (MSKCC) criteria. According to the Turin proposal, also endorsed by the WHO classification system, papillary-like nuclear features must be absent, the growth pattern is solid/trabecular/insular, and tumors should present at least one feature among high mitotic index, necrosis and convoluted nuclei [25]. The MSKCC criteria are less rigid; they are based on the mere presence of a high mitotic rate and/or tumor necrosis, thus PDTCs are diagnosed independently of the observed growth pattern and of PTC nuclear changes [26]. Depending on which diagnostic system is adopted, differences have been reported not only concerning the prevalence and the prognosis of PDTC, but also affecting their molecular status. In fact, Turin-PDTCs harbor more frequently *RAS* mutations, while MSKCC-PDTC are enriched with *BRAF* mutations [27]; moreover, Turin-PDTCs compared to MSKCC-PDTCs show a higher incidence of *TP53*, *EIF1AX*, *PTEN* and *PIK3CA* mutations [28].

ATC is a highly aggressive tumor, with distant metastases at diagnosis in 30–40% of cases [2]. At microscopic examination, it has a variable cellular appearance, with a complete loss of follicular differentiation. ATC is often associated with a coexisting DTC, or with a clinical history of DTC, and this suggests that clones from DTC may undergo a morphological evolution towards dedifferentiation [29]. In this context, molecular studies have shown partial overlapping between ATCs and their differentiated counterparts [30].

Recently, many authors have performed massive parallel sequencing studies on both PDTCs and ATCs to investigate in detail their molecular aspects and to find differences between these two tumors. The frequency of the main mutations and fusions detected in PDTCs and ATCs are reported in Table 1.

Considering the differences between PDTCs and ATCs, it has been reported that ATCs show significantly higher frequencies of *TP53*, *TERT* promoter, *PIK3CA* and *PTEN* mutations compared to PDTCs [30,31]. Moreover, ATCs also harbor *ATM*, *NF1*, *NF2*, *CDKN2A*, *CDKN2B* and *RB1* mutations [5,30,32,33,37]. On the other hand, PDTCs more frequently display gene fusions (*RET*, *ALK*, *NTRK1*, *NTRK3*) compared to ATCs [30,31].

## 4. Molecular Alterations in Advanced Differentiated Cancers

The study of WDTCs with clinically aggressive behavior is crucial to understand whether specific molecular alterations could be indicative of high-risk tumors. Moreover, patients with advanced treatment-resistant tumors should be investigated for the presence of druggable alterations. The definition of “advanced” is not univocal: Table 2 summarizes the main findings so far reported on advanced PTCs and FTCs, including cases with persistent/recurrent disease, radioiodine resistant tumors, stage IV at presentation, distant metastases.

By comparing the mutational landscape of 139 advanced PTCs with TCGA results, Chen reported that the co-occurrence mutation rate was significantly higher in advanced PTCs (7% versus 2.5% in TCGA cases); similarly, *PIK3CA* and *TP53* mutations were significantly more frequent in advanced PTCs [35]. Furthermore, as reported in Table 2, advanced PTCs harbor *TERT* promoter mutations more frequently than non-advanced tumors (about 48% versus 10–15% [18]), with an incidence even higher than that observed in PDTCs (22–40%); the frequency in advanced FTCs was even comparable to that of ATC (66% versus 68%). These results confirm the crucial role of *TERT* promoter mutations in thyroid cancer dedifferentiation and progression.

## 5. Molecular Markers for Targeted Therapy in Thyroid Cancer

The advances in the field of biological drugs have led to the development of targeted agents against specific molecular alterations. *BRAF* inhibitors alone or in combination with MEK inhibitors have shown good response rates in *BRAF*^V600E^ ATCs [41]. The administration of multi-target thyrosine kinase inhibitors (TKI) such as sorafenib and lenvatinib, approved in many countries, has improved the progression-free survival of radioiodine-resistant DTCs [42,43]. Indeed, a recent meta-analysis reports that treatment with lenvatinib in DTC patients achieved a pooled partial response rate of 69%, and a progression-free survival of 19 months [44]. However, prognosis in ATC patients remained poor even with TKI therapy (pooled progression-free survival was 5 months), and a complete response was rarely achieved (0.3%). Moreover, owing to its inhibitory effects against multiple targets, TKI treatment causes adverse events involving fatigue, gastrointestinal symptoms, hypertension, liver disfunction and affecting also thyroid function and metabolism [45].

With regard to immunotherapy, it has been demonstrated that ATCs express PD-L1, but evidences of the efficacy of immune check-point inhibitors in the treatment of thyroid cancer patients are still limited [29,46]. The use of the anti-PD-1 drug pembrolizumab in combination with lenvatinib is currently being evaluated in clinical trials enrolling DTC, PDTC and ATC patients (NCT02973997; NCT04731740).

Drugs targeting gene rearrangements involving thyrosine kinase receptors such as *NTRK* have been FDA-approved for *NTRK*-fused cancers, independently of the tumor type. In particular, larotrectinib selectively targets *NTRK* rearrangements, while entrectinib has effects also on *ALK*- and *ROS1*-altered receptors [47,48]. Preliminary data on thyroid cancer show that larotrectinib is effective, demonstrating an overall response rate of 75% [49]. In spite of the rarity of *NTRK* fusions in non-pediatric thyroid cancers (2–5%) [9,10], these targeted drugs might represent a promising strategy of treatment in advanced tumors lacking the most common molecular alterations.

Finally, FDA has recently approved two selective *RET* inhibitors: selpercatinib and pralsetinib. These drugs have been evaluated on *RET*-altered solid cancers, and approved for mutated MTC, and rearranged DTC and non-small cell lung cancer. Considering only the *CCDC6/RET*-positive DTCs (also known as RET/PTC1 fusion) enrolled in both clinical trials, the overall response rate for selpercatinib and pralsetinib was 100% (8/8 patients) and 83% (5/6 patients), respectively [50]. *RET* fusions in ATC are quite uncommon (see Table 1), but these results have paved the way to additional therapeutic options for a non-negligible portion of patients with advanced DTC and PDTC.

## 6. Copy Number Alterations, Gene Expression and microRNA in Thyroid Cancer

Alterations other than gene mutations and fusions have been investigated in thyroid cancer. However, their application in the clinical context is less straightforward and may require complex data analysis as well as procedure standardization.

The biological and clinical role of copy number alterations (CNA) in thyroid cancer has been scarcely investigated, probably due to the technical difficulties related to this type of analysis. Somatic CNAs are characterized by the loss or gain of one or more copies of a gene or a locus in tumor cells. In TCGA cohort, 27% of tumors had CNAs, and this group was enriched in PTCs lacking driver mutations or fusions [9]. These findings indicate that somatic CNAs can represent driver genetic events in PTC tumorigenesis. The wide spread of massive parallel sequencing platforms and the availability of targeted panels that also include CNA analysis are likely to expand knowledge in this field of study.

The expression of specific transcripts and/or microRNAs (miRNA) has been proposed for diagnostic and prognostic purposes. There are currently no mRNA transcripts that have proved sensitive or specific enough to be applied as stand-alone markers in routine applications. Thanks to the advent of multitarget platforms (e.g., the nCounter nanoString platform), the most recent scientific approaches have been investigating gene panels able to identify peculiar expression patterns, rather than single markers [51]. This analysis strategy, in conjunction with mutation analysis, is applied to the differential diagnosis of indeterminate thyroid nodules by diagnostic commercial tests such as the Afirma Genomic Sequencing Classifier and the Thyroseq v3 Genomic Classifier [52].

MiRNAs are small non-coding RNA molecules that regulate the expression of specific transcripts in an epigenetics manner. Several miRNAs have been reported as being involved in thyroid cancer pathogenesis as well as in cancer progression. Some of them (for example miR-146b, miR-221 and miR-222) have been consistently and widely reported as up-regulated in PTC by many authors, and could therefore serve as PTC markers [53,54,55]. It has been demonstrated that up-regulated miRNAs target suppressor genes, such as *PTEN*, belonging to the MAPK and the PI3K/AKT pathways [54]. On the contrary, suppressor miRNAs have been frequently reported as down-regulated in thyroid cancer (for instance miR-375, miR-7 and miR204) [55,56]. Moreover, since miRNAs are differentially expressed in thyroid tumors versus benign lesions and in different histotypes of thyroid cancer, the development of miRNA-based molecular test is an appealing strategy for the diagnostic and prognostic definition of thyroid tumors [57].

## 7. Discussion

Molecular markers in thyroid pathology can be considered as having different levels of usability. While the diagnostic role of mutational testing on thyroid tumor tissue is limited, in thyroid cytology molecular markers support differential diagnosis between benign and malignant lesions in indeterminate nodules, thus playing the role of diagnostic markers. In this context, the detection of *BRAF*^V600E^ mutation indicates a malignancy with high confidence. In indeterminate thyroid nodules, however, the frequency of *BRAF*^V600E^ mutations should be low, because *BRAF*-like tumors are generally easily recognizable on cytology [58]. The detection of *RAS* mutations probably requires an integration with clinical information (e.g., ultrasound features, nodule size, patient age and sex). *RAS* mutations can indeed be present also in benign nodules, identifying a “surgical disease” in no more than 75% of cases [59]. Considering Hürthle cell nodules, the differential diagnosis of benign and malignant neoplasms represents an issue that cannot be easily solved by molecular testing, for at least two main reasons: (1) these lesions harbor genetic alterations such as chromosome aneuploidy, which are not routinely searched in thyroid cytology, and are difficult to analyze; (2) it is still unclear to what extent these molecular alterations are specific for malignancy.

An interesting point is to understand how mutations labelling aggressive tumors could serve as prognostic markers in cytology. For instance, the search for *TERT* promoter mutations in suspicious and malignant cytology could identify nodules at high risk of advanced histology or dedifferentiation [60]. Similarly, alterations in the *TP53* gene are rarely found in WDTCs, but their frequency increases in subgroups of advanced, high-risk WDTCs [5]. Therefore, *TP53* screening in suspicious and malignant thyroid nodules would be informative only in a few cases, thus not proving to be cost-effective. It would be necessary to establish whether specific cyto-morphological characteristics might help in an early selection of nodules for which an in-depth molecular analysis could be worthwhile.

**Figure 1:**
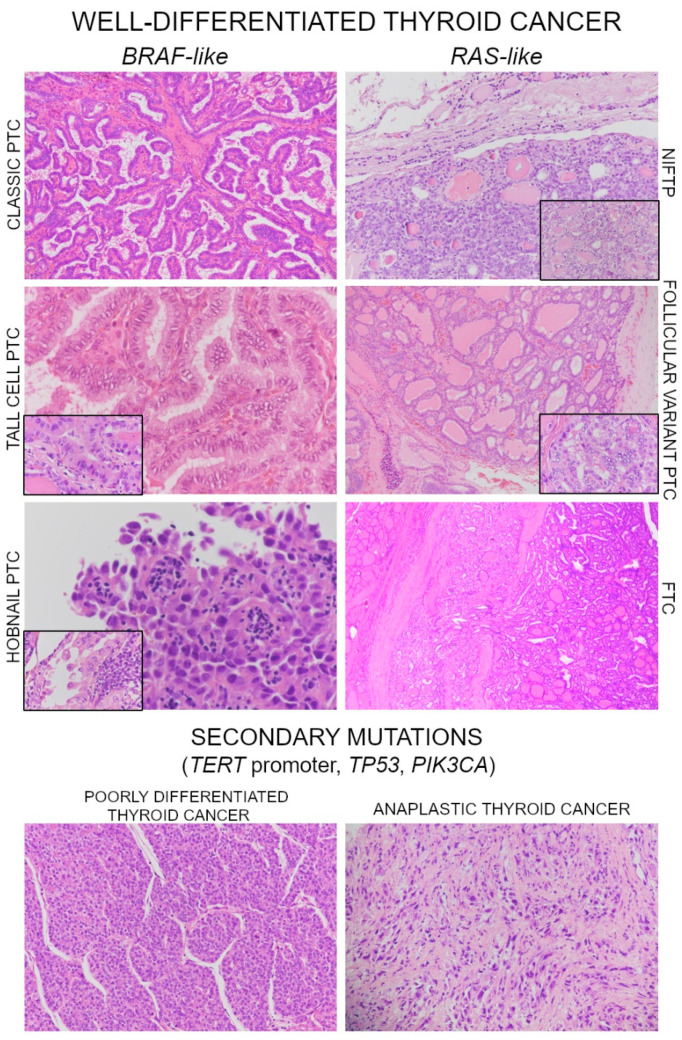
Examples of *BRAF*-like and *RAS*-like well-differentiated thyroid tumors. Original magnification of images is reported in brackets. In particular, the image shows, on the left: classic papillary thyroid carcinoma (PTC, 10×), tall cell variant PTC (40×, insect 60×) and hobnail variant PTC (40×, insect 60×); on the right: noninvasive follicular neoplasm with papillary-like nuclear features (NIFTP, 20×, insect 40×), follicular variant PTC (10×, insect 60×) and a minimally invasive follicular thyroid carcinoma (FTC, 2×). The acquisition of secondary mutations leads to dedifferentiation towards poorly differentiated (insular pattern, image on the bottom, left, 20×) and anaplastic thyroid cancer (image on the bottom, right, 20×). The insets represent details of specific diagnosis-related aspects of tumors: the taller-than wide shape of cells is showed in more detail for tall cell variant PTC; cells with atypically bulged nuclei are showed for the hobnail variant PTC; the presence of PTC nuclear features is showed in detail for the NIFTP and the follicular variant PTC, where the follicular architecture alone is not informative.

Considering the prognostic utility of molecular testing on tumor tissue, the TGCA study has demonstrated clearly that the molecular landscape of PTC is relatively simple, with an overall low mutational load and mutational events recurring in few genes [9]. Therefore, routinely performed molecular testing in all WDTCs would add prognostically useful information in a limited fraction of cases. On the other hand, this review of the literature has shown that advanced PTCs and FTCs have an incidence of secondary mutations that is halfway between WDTCs and PDTC/ATCs. This indicates a progressive accumulation of oncogenic alterations that lead to loss of differentiation, thus highlighting the importance of investigating tumors for the presence of high-risk mutations, such as *TERT* promoter, *PIK3CA*, and *TP53* mutations (Figure 1).

Mutational testing is not routinely recommended by the current guidelines of the American Thyroid Association; however, it has been acknowledged that markers such as *BRAF*, *TERT*, and *TP53* can help in refining the risk stratification of patients with DTC [61].

Biologically, while driver mutations may cause tumor initiation and proliferation, the deleterious effect of secondary mutations consists in a progressive disruption of signaling pathways, leading to a loss of differentiation. For instance, *TP53* is an important tumor suppressor gene responsible for cell cycle arrest in case of cell damage. Its loss, caused mainly by inactivating mutations, leads to the impairment of this protective mechanism [62]. As mentioned above, *TERT* promoter mutations promote telomerase activation; in addition, a synergistic effect with driver mutations, mainly *BRAF*, in promoting tumor aggressiveness has been demonstrated [20]. The specific role of *PIK3CA* mutations coexisting with other driver mutations in thyroid cancer has been poorly investigated. However, it is known that a cooperation between MAPK and PI3K/AKT pathways can exist, thus the activation of both pathways should have a favorable effect on tumor growth and progression [63].

Alongside the prognostic role of secondary mutations in WDTC, advanced tumors should also be tested for predictive biomarkers of response to therapy. In the setting of advanced and dedifferentiated thyroid cancer, the testing of genetic alterations in genes other than *BRAF* and *RAS* (virtually, easily evaluable in all molecular pathology laboratories) is acquiring an increasing importance. In particular, the detection of rearrangements in *NTRK*, *RET*, and *ALK* genes makes patients eligible for treatment with newly approved targeted drugs, whose efficacy has proved to be extremely promising.

## 8. Conclusions

The genetic hallmarks of follicular-derived thyroid cancer are highly variable depending on specific tumor histotypes. While the majority of WDTC have a simple genetic background, with recurrent mutations in *BRAF* and *RAS* genes, poorly differentiated and anaplastic cancers are often characterized by multiple co-occurring mutational events. The advanced WDTC show a rate of secondary mutations which is similar to that of less differentiated forms, thus highlighting the importance of mutational testing in identifying high-risk tumors. Moreover, the knowledge of the molecular status of a tumor not only integrates clinical and pathological information, but can also guide targeted-based therapies.

## Figures and Tables

**Table 1:** Most frequent molecular alterations reported in PDTC and ATC [5,14,30,31,32,33,34,35,36].

Gene	PDTC			ATC		
	n° Mutant/n° Total	Frequency Range	Pooled Frequency	n° Mutant/n° Total	Frequency Range	Pooled Frequency
*BRAF*	57/220	15–33%	26%	166/395	20–56%	42%
*RAS*	48/220	9–39%	22%	100/395	20–33%	25%
*EIF1AX*	11/125	5–11%	9%	22/181	8–14%	12%
*PIK3CA*	15/220	2–20%	7%	65/395	9–44%	16%
*PTEN*	6/220	4–33%	3%	45/395	11–20%	11%
*TERT*	43/125	22–40%	34%	242/355	56–75%	68%
*TP53*	45/220	8–67%	20%	244/395	25–80%	62%
*RET* fusion	11/125	6–15%	9%	5/355	0–2%	1%
*PPARG* fusion	4/125	2–4%	3%	0/159	0%	0%
*ALK* fusion	4/125	2–4%	3%	0/355	0%	0%
*NTRK* fusion	1/41	0–2%	2%	5/322	1–4%	2%

Abbreviations: PDTC, poorly differentiated thyroid cancer; ATC, anaplastic thyroid cancer.

**Table 2:** Gene mutations and rearrangements described in advanced well-differentiated thyroid carcinomas [5,35,37,38,39,40].

Gene	Advanced PTC			Advanced FTC		
	n° Mutant/n° Total	Frequency Range	Pooled Frequency	n° Mutant/n° Total	Frequency Range	Pooled Frequency
*BRAF*	583/894	45–71%	65%	6/136 ^1^	0–8%	4%
*RAS* ^2^	68/890	1–23%	8%	83/136	8–90%	61%
*EIF1AX*	3/62	0–10%	5%	5/88	0–40%	6%
*PIK3CA*	36/669	3–6%	5%	2/100	0–3%	2%
*PTEN*	10/669	0–2%	1%	9/100	0–14%	9%
*TERT*	314/651	13–62%	48%	68/103	50–82%	66%
*TP53*	64/669	3–13%	10%	9/100	0–12%	9%
*RET* fusion	37/558	3–7%	7%	0/89	0%	0%
*PPARG* fusion	0/59	0%	0%	0/89	0%	0%
*ALK* fusion	3/527	<1–2%	1%	0/89	0%	0%
*NTRK* fusion	8/527	1–5%	2%	0/89	0%	0%
*BRAF* fusion	14/527	0–3%	3%	0/89	0%	0%

Abbreviations: PTC, papillary thyroid cancer; FTC, follicular thyroid cancer. ^1^ Five out of 6 were non-V600E mutations; considering *BRAF*^V600E^ only, the pooled frequency was equal to 0.7%. ^2^ Some authors evaluated the *NRAS* gene only.
